# An exopolysaccharide pathway from a freshwater *Sphingomonas* isolate

**DOI:** 10.1128/jb.00169-24

**Published:** 2024-07-15

**Authors:** Alexandra G. Goetsch, Daniel Ufearo, Griffin Keiser, Christian Heiss, Parastoo Azadi, David M. Hershey

**Affiliations:** 1 Department of Bacteriology, University of Wisconsin–Madison, Madison, Wisconsin, USA; 2 Complex Carbohydrate Research Center, University of Georgia, Athens, Georgia, USA; Philipps-Universitat Marburg Fachbereich Biologie, Marburg, Germany

**Keywords:** *Sphingomonas*, polysaccharide, *wzy*, biopolymer

## Abstract

Bacteria embellish their cell envelopes with a variety of specialized polysaccharides. Biosynthesis pathways for these glycans are complex, and final products vary greatly in their chemical structures, physical properties, and biological activities. This tremendous diversity comes from the ability to arrange complex pools of monosaccharide building blocks into polymers with many possible linkage configurations. Due to the complex chemistry of bacterial glycans, very few biosynthetic pathways have been defined in detail. As part of an initiative to characterize novel polysaccharide biosynthesis enzymes, we isolated a bacterium from Lake Michigan called *Sphingomonas* sp. LM7 that is proficient in exopolysaccharide (EPS) production. We identified genes that contribute to EPS biosynthesis in LM7 by screening a transposon mutant library for colonies displaying altered colony morphology. A gene cluster was identified that appears to encode a complete *wzy/wzx-*dependent polysaccharide assembly pathway. Deleting individual genes in this cluster caused a non-mucoid phenotype and a corresponding loss of EPS secretion, confirming the role of this gene cluster in polysaccharide production. We extracted EPS from LM7 cultures and determined that it contains a linear chain of 3- and 4-linked glucose, galactose, and glucuronic acid residues. Finally, we show that the EPS pathway in *Sphingomonas* sp. LM7 diverges from that of sphingan-family EPSs and adhesive polysaccharides such as the holdfast that are present in other *Alphaproteobacteria*. Our approach of characterizing complete biosynthetic pathways holds promise for engineering polysaccharides with valuable properties.

**IMPORTANCE:**

Bacteria produce complex polysaccharides that serve a range of biological functions. These polymers often have properties that make them attractive for industrial applications, but they remain woefully underutilized. In this work, we studied a novel polysaccharide called promonan that is produced by *Sphingomonas* sp. LM7, a bacterium we isolated from Lake Michigan. We extracted promonan from LM7 cultures and identified which sugars are present in the polymer. We also identified the genes responsible for polysaccharide production. Comparing the promonan genes to those of other bacteria showed that promonan is distinct from previously characterized polysaccharides. We conclude by discussing how the promonan pathway could be used to produce new polysaccharides through genetic engineering.

## INTRODUCTION

Bacterial cells are decorated with elaborate arrays of carbohydrates (1). Polysaccharides such as capsules, O-antigens, and exopolysaccharides (EPSs) serve as a physical interface between the surface of the bacterium and other objects in the environment (2, 3). These glycans have a tremendous influence on fitness, and their properties have specialized to reflect the wide range of niches colonized by bacteria (4). Immunomodulators (5), adhesins (6), and gelling agents (7) are among the many classes of bacterial polysaccharides that have been identified. Collectively, these polymers present a rich source of chemical diversity that can be utilized for human benefit.

Bacteria synthesize complex polysaccharides using the *wzx*/*wzy* strategy. The early stages of *wzx*/*wzy*-dependent polysaccharide biosynthesis require an initiating sugar transfer enzyme and a series of glycosyltransferase (GT) enzymes that attach monosaccharides sequentially onto a lipid carrier at the cytoplasmic face of the inner membrane. The resulting glycolipid intermediate is called the repeating unit. A highly conserved set of assembly proteins flips (Wzx) repeating units across the cytoplasmic membrane, polymerizes (Wzy) them into a chain, and secretes (Wzc and Wza) the mature glycan into the extracellular space (8). The sugar composition and linkage configurations present in *wzy*-dependent polysaccharides vary widely. Each biosynthetic pathway utilizes a unique set of GTs to assemble a repeating unit oligosaccharide containing three to eight sugars (9). Variability in substrate and linkage specificities among GT enzymes gives rise to the vast chemical diversity of bacterial glycans.

Bacterial polysaccharides have the potential to be used in a range of applications, but few have found widespread use. Current production pipelines rely on extracting polysaccharides directly from producing organisms (10–12). This approach restricts the potential of glycans that are produced at lower quantities or by organisms that are not amenable to large-scale fermentation. We are interested in developing an alternative approach whereby bacteria are genetically modified to produce polysaccharides recombinantly. Not only would this strategy allow for the over-production of low-abundance glycans and facilitate the characterization of biosynthesis pathways from genetically intractable organisms, but it could also provide a framework for engineering novel polysaccharides through combinatorial biosynthesis. For instance, the sugar compositions of natural glycans could be modified by exchanging genes for GTs with different substrate specificities. Such engineering efforts require a pool of glycan assembly enzymes with well-characterized specificities that can be used to introduce chemical complexity.

Biosynthetic pathways for *wzx*/*wzy-*dependent polysaccharides have seldom been characterized in detail. Capsular polysaccharide A from *Bacteroides fragilis* (13)*,* succinoglycan from *Sinorhizobium meliloti* (14)*,* xanthan gum from *Xanthomonas campestris* (15), and colonic acid from *Escherichia coli* (16) represent rare examples for which the enzymatic steps of repeating unit assembly have been clearly defined. For each of these glycans, a clear biomedical, industrial, or agricultural relevance provided justification for the considerable effort required to elucidate the biosynthetic pathways. However, these model pathways encompass only a minuscule fraction of the chemical diversity seen in bacterial glycans (17), and polysaccharides with promising properties continue to be identified (18–20). Defining additional biosynthetic pathways for bacterial polysaccharides will lead to the discovery of novel enzymatic functionalities.

To search for novel polysaccharide biosynthesis pathways, we began isolating bacteria from freshwater lakes that were proficient in polysaccharide production. We describe here an isolate from Lake Michigan called *Sphingomonas* sp. LM7. LM7 displays a mucoid colony morphology on agar plates that is indicative of EPS production. We utilized a genetic screen based on colony morphology to identify genes involved in polysaccharide biosynthesis. Two genetic loci emerged as key determinants of colony morphology. One locus contains genes for producing a secreted polysaccharide that we have named promonan, and the other contains genes for producing a second polysaccharide that remains associated with the cell surface. We defined the monosaccharide composition of promonan, performed a detailed analysis of genes involved in its biosynthesis, and compared the promonan genes to those in other EPS pathways. Our results indicate that the assembly of the promonan repeating unit differs substantially from polysaccharides such as sphingans and holdfasts that are produced by related bacteria. We conclude that the promonan EPS belongs to a novel family of *wzx*/*wzy-*dependent polysaccharides, which could serve as a valuable scaffold for producing engineered glycans.

## RESULTS

### Isolation and phenotypic characterization of *Sphingomonas* sp. LM7

**Fig 1:**
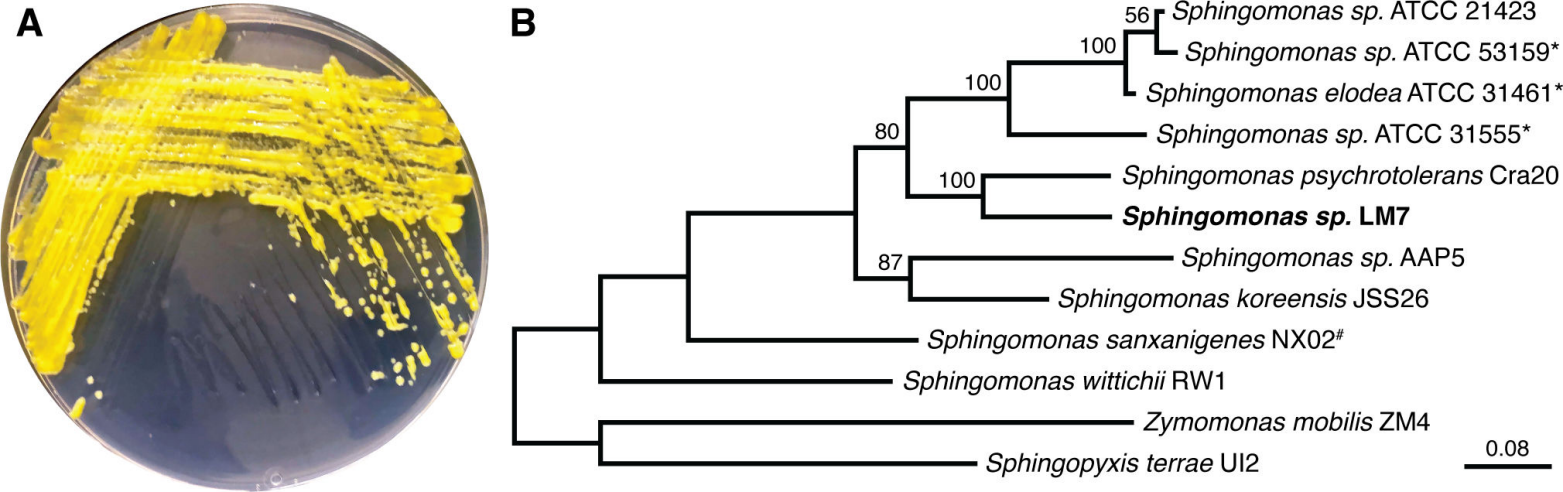
Isolation of *Sphingomonas* sp. LM7. (**A**) *Sphingomonas* sp. LM7 growing on agar plates. The isolate grows as yellow, mucoid colonies. (**B**) Phylogenetic tree showing select isolates from the *Sphingomonadales* order of *Alphaproteobacteria*. The neighbor-joining tree was constructed from 16S rDNA sequences aligned with ClustalX. Bootstrapping values are indicated at the relevant nodes. * indicates canonical sphingan-producing strains. # highlights the non-canonical sphingan called sanxan produced by *Sphingomonas sanxanigenes* NX02. Note that the sanxan biosynthesis genes appear distinct from the sphingan or promonan genes (22).

We collected water from Lake Michigan at Promontory Point in Chicago, IL, USA, and serially diluted it on PYE agar (21). LM7 was isolated based on its colony morphology on agar plates. The organism grew as “mucoid” yellow colonies that displayed a shiny, gelatinous appearance (Fig. 1A). This mucoid phenotype was more pronounced when the bacterium was incubated at lower temperatures and on agar plates supplemented with sugars such as glucose or sucrose. We predicted that LM7 was secreting a novel polysaccharide because the mucoid colony morphology is often associated with EPS production.

Sequencing of the 16S rRNA gene placed LM7 in the genus *Sphingomonas,* with the most similar pure culture isolate being *Sphingomonas psychrotolerans* Cra20 (95% identity) (23). Certain members of the genus *Sphingomonas* produce EPSs known as sphingans (gellan, welan, and diutan) (24), and we were curious if LM7 was producing a sphingan-type polysaccharide. We sequenced the genome of the LM7 and found that it contains a single, circular chromosome of 4,155,514 bases. There are two 16S rRNA genes with identical sequences. We constructed a phylogenetic tree based on the 16S rRNA gene sequences of several isolates within the order *Sphingomonadales*. LM7 and *S. psychrotolerans* form a clade that is distinct from canonical sphingan-producing strains (Fig. 1B). We were also unable to identify loci in the LM7 genome that resemble the *spn* genes for sphingan biosynthesis (25) or the sanxan biosynthesis genes from *Sphingomonas sanxanigenens* NX02 (22). We predicted that *Sphingomonas* sp. LM7 was producing a novel polysaccharide that is distinct from previously characterized EPSs.

### Identification of genes affecting colony morphology

**Fig 2:**
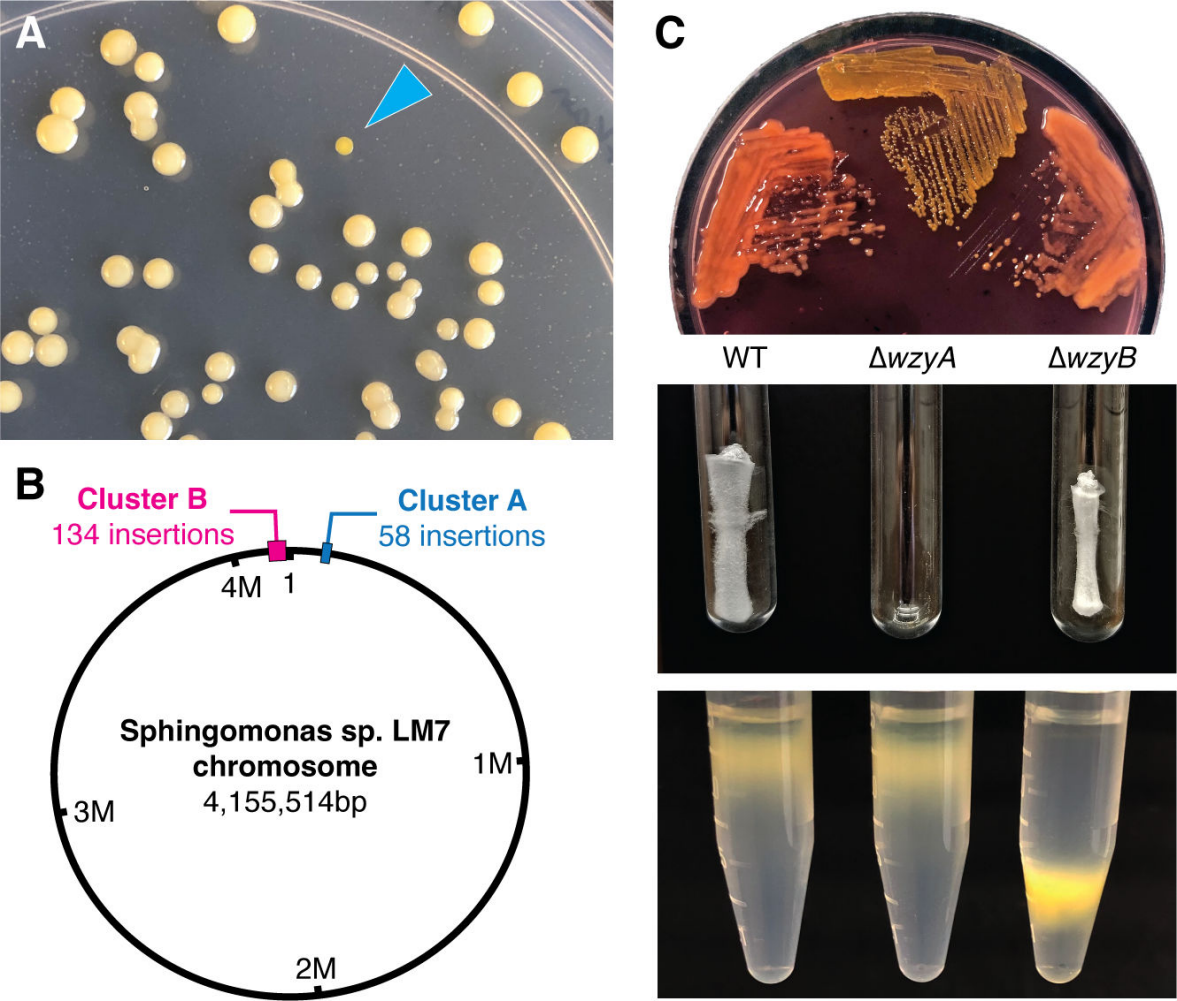
Identification of two polysaccharide biosynthesis clusters in LM7. (**A**) Screening for transposon insertion mutants with altered colony morphology. Mutagenized cells were plated on agar plates. Under these conditions, the majority of colonies showed mucoid morphology. The blue carat points to a non-mucoid mutant. (**B**) Map of the LM7 genome showing the locations of the two gene clusters that influence colony morphology. (**C**) Phenotypes of ∆*wzyA* and ∆*wzyB* mutants. The top image shows the growth of LM7 strains on a solid medium containing Congo Red dye. Wild type and the ∆*wzyB* mutant appear mucoid and react with the Congo Red dye, while the ∆*wzyA* mutant does not. The middle image shows an EPS extraction. Wild-type and ∆*wzyB* cultures produce secreted matrix. ∆*wzyA* cells do not secrete this matrix. The bottom image shows the density gradient centrifugation of LM7 strains. The ∆*wzyB* mutant displays increased cell density relative to wild-type and ∆*wzyA* cells.

We performed a genetic screen to study the molecular basis for EPS production in LM7. A transposon mutant library was plated on a defined medium supplemented with glucose. The overwhelming majority of transposon insertion mutants showed the mucoid phenotype under these conditions. We screened the mutants for colonies that have a non-mucoid, “matte” appearance (Fig. 2A). Two hundred fifty individual mutants with altered colony morphology were isolated, and the transposon insertion sites were mapped by arbitrary PCR. The insertion sites are summarized in Table 1.

**TABLE 1:** Sites of transposon insertions affecting colony morphology in LM7

Gene	Frequency	Annotation
RS00295	1	PAS domain-containing methyl-accepting chemotaxis protein
RS00350^*a*^	7	WcaJ family polyisoprenylphosphate hexose-1-phosphate transferase
Upstream RS00360^*a*^	1	Predicted promoter region of BXU08_RS00360
RS00360^*a*^	4	Beta-barrel outer membrane porin
RS00365^*a*^	2	Wza family outer-membrane polysaccharide export protein
RS00370^*a*^	2	AAA+ family ATPase
RS00375^*a*^	4	Wzz family polysaccharide chain length determinant
RS00380^*a*^	3	P-loop NTPase
RS00385^*a*^	4	WecG/TagA family glycosyltransferase
RS00395^*a*^	5	Wzy family polysaccharide polymerase
RS00400^*a*^	1	GT2 family glycosyltransferase
RS00405^*a*^	1	GT2 family glycosyltransferase
RS00410^*a*^	6	GT2 family glycosyltransferase
RS00415^*a*^	2	GH10 family glycosylhydrolase
RS00420^*a*^	9	OafA family acyltransferase
RS00425^*a*^	4	GT2 family glycosyltransferase
RS00430^*a*^	10	GH16 family glycosylhydrolase
RS00665	2	DedA family membrane protein
RS02105	2	PAS domain-containing sensor histidine kinase
RS06730	1	Tryptophan halogenase
RS07760	1	Type II secretion system protein GspL
RS07770	1	Type II secretion system protein GspH
UpstreamRS09000	1	Predicted promoter region of BXU08_RS09000
RS09000	2	OmpR family response regulator
RS10090	1	Hypothetical protein
RS10945	1	Acetoacetyl-CoA reductase PhbB
RS11025	2	Poly(R)-hydroxyalkonic acid synthase PhaC
RS12915	1	Helix-turn-helix family protein
RS13595	1	FliA family transcriptional regulator
RS14850	1	Bifunctional (p)ppGpp synthase-hydrolase SpoT
RS15820	1	UDP-Galactose 4-epimerase GalE
RS18205	1	GT2 family glycosyltransferase
RS18930	2	Major facilitator superfamily transporter
UpstreamRS19155^*b*^	1	Predicted promoter region of BXU08_RS19155
RS19155^*b*^	3	Bifunctional GGDEF-EAL diguanylate cyclase-hydrolase
RS19160^*b*^	6	TPR domain-containing sensor histidine kinase
DownstreamRS19160^*b*^	3	Insertion downstream of BXU08_RS19160
RS19205^*b*^	1	NAD(P)+-dependent nucleotide sugar dehydrogenase
RS19215^*b*^	12	Amidotransferase, exosortase associated
RS19220^*b*^	18	PLP-dependent aminotransferase
RS19225^*b*^	8	WxcM family acetyltransferase
RS19230^*b*^	8	MviM family dehydrogenase
RS19235^*b*^	14	GT4 family glycosyltransferase
RS19250^*b*^	8	SDR family NAD(P)+-dependent oxidoreductase
RS19260^*b*^	12	WbuX family N-acetyl sugar amidotransferase
RS19265^*b*^	10	AlgZ/HisF2 family acetamidino modification system
RS19270^*b*^	9	HisH family amidotransferase subunit
RS19290^*b*^	2	EpsG family glycosyltransferase
RS19295^*b*^	12	GT4 family glycosyltransferase
RS19300^*b*^	2	Wzy family polysaccharide polymerase
RS19310^*b*^	2	Wzc family tyrosine autokinase
RS19360^*b*^	3	NAD(P)+-dependent epimerase

^*a*^ Insertion sites within the boundaries of Cluster A (promonan).

^*b*^ Insertion sites within the boundaries of Cluster B (cell-associated polysaccharide).

Of the 250 mutants, 6 contained insertions in intergenic regions, while the remaining 244 mapped to coding regions. Forty-eight open reading frames (ORFs) and four intergenic regions were disrupted. Insertion sites were scattered across the chromosome, but the majority of the mutations (192 of 250) were concentrated at two loci (Fig. 2B). The first locus (Cluster A in Fig. 2B) contained 58 insertions. It spans ~18.5 kb and encodes a gene cluster that contains 17 open reading frames. The second locus (Cluster B in Fig. 2B) is separated from Cluster A by ~102 kb. One hundred thirty-four mutations were identified in Cluster B, which spans a nearly 48 kb region encompassing over 40 open reading frames. We conclude that the genetic determinants of colony morphology in *Sphingomonas* sp. LM7 are concentrated at two separate loci in the genome.

### Two polysaccharide biosynthesis gene clusters

Each of the two loci that affect colony morphology in LM7 contains genes with homology to *wzy, wzx, wzz,* and *wza* along with a suite of GT genes, suggesting that two distinct polysaccharides influence colony morphology under our growth conditions. To differentiate the roles of Cluster A and Cluster B in polysaccharide production, we generated in-frame deletions (∆*wzyA* and ∆*wzyB*) of the genes predicted to encode the Wzy polymerase enzyme from each cluster. We found that including Congo Red dye in agar plates accentuated the mucoid phenotype by causing wild-type LM7 colonies to take on a reddish tint. The ∆*wzyA* mutant displayed a rough texture on Congo Red plates and remained yellow in appearance, indicating that this mutant did not produce a Congo Red reactive matrix. The ∆*wzyB* mutant appeared indistinguishable from the wild-type strain on Congo Red plates. The colonies remained mucoid and took on the reddish hue of the Congo Red dye (Fig. 2C). These results indicate that LM7 produces a Congo Red reactive matrix that requires the *wzyA* gene but not the *wzyB* gene.

LM7 secretes a gelatinous matrix into a liquid medium during growth that can be precipitated with ethanol. This material is resistant to treatment with protease and DNase and remains in the aqueous phase during phenol fractionation. We subjected spent medium from the wild-type, ∆*wzyA,* and ∆*wzyB* strains to an extraction that had been optimized for the isolation of this secreted matrix. Lyophilized extracts prepared from wild-type LM7 cultures contained a white, fibrous material reminiscent of cotton. The fibrous material was completely absent in extracts prepared from the ∆*wzyA* mutant, but the ∆*wzyB* mutant produced extracts that were indistinguishable from the wild type. Thus, *wzyA,* but not *wzyB,* is required for the secretion of the gelatinous material (Fig. 2C).

Some of the transposon mutants isolated in our initial screen appeared to sediment differently during centrifugation. We predicted that differences in the appearance of cell pellets reflected changes in the density of the cell envelope. To investigate cell densities, we subjected our strains to density gradient centrifugation in a solution of Percoll. Wild-type cells remained near the top of the gradient under our centrifugation conditions, and the ∆*wzyA* mutant retained the buoyancy of the wild-type strain. ∆*wzyB* cells sedimented further along the gradient than wild-type cells, indicating that this mutation causes the cell envelope to become denser (Fig. 2C). We conclude that *wzyB* is required for producing a polysaccharide that increases the buoyancy of the LM7 cell envelope.

Our analysis of the ∆*wzyA* and ∆*wzyB* mutants is consistent with a model in which LM7 produces two distinct polysaccharides. Most of the colony morphology mutants we isolated contained transposon insertions in one of two regions of the genome, and each of these loci appears to encode a complete set of genes for producing a *wzx*/*wzy-*dependent polysaccharide. Deleting the *wzy* gene, which codes for the repeating unit polymerase enzyme, from the two gene clusters caused disparate phenotypes. The ∆*wzyA* mutation disrupted the LM7’s ability to secrete a gelatinous, Congo Red reactive EPS, while the ∆*wzyB* mutant displayed a denser cell envelope. We conclude that Cluster A is responsible for the production of a secreted polysaccharide and that Cluster B contains the genes for a cell surface-associated polysaccharide.

### Isolation and analysis of a secreted polysaccharide

We developed a method to purify the secreted EPS from LM7. Growth at lower temperatures (18°C) and supplementing the growth medium with glucose (2%) led to enhanced EPS secretion. EPS could be precipitated from the spent medium of LM7 cultures with alcohol and subsequently enriched with a liquid-liquid extraction using aqueous phenol. After dialysis and lyophilization, the resulting extracts contained a patch of fluffy, white fibers. When the extraction was carried out with ∆*wzyA* cultures, only trace amounts of an oily residue were observed (Fig. 2C). Thus, our extraction leads to the purification of a secreted polysaccharide that requires the *wzyA* gene. We have named this polysaccharide promonan after the site on Promontory Point in Chicago, IL, USA, from which LM7 was isolated.

Promonan extracts were subjected to a series of chemical analyses to characterize the polysaccharide’s chemical composition. Analysis of *O-*trimethylsilyl (TMS) methyl glycoside derivatives prepared from promonan showed that glucose, glucuronic acid, and galactose were the major monosaccharide constituents of promonan (Table 2). The exact ratios of the three sugars could not be determined from this analysis, but glucose was clearly more abundant than the other two residues. We studied the linkage patterns in promonan using a recently developed method for analyzing partially methylated alditol acetate derivatives (PMAAs) derived from uronic acid sugars (26). All three monosaccharides (Glc, GlcA, and Gal) were present in 3- and 4-linked forms (Table 3). Trace amounts (≤1% of total) of terminal and multiple-linked forms of each monosaccharide were also detected. Our analysis indicates that promonan is composed of glucose, glucuronic acid, and galactose. It also suggests that the repeating unit comprises a linear chain containing 3- and 4-linked forms of each residue.

**TABLE 2:** Monosaccharide composition of promonan EPS^*a*^

Glycosyl residue	Estimated mol%
Glucuronic acid (GlcA)	23.9
Galactose (Gal)	16.5
Glucose (Glc)	59.6

^*a*^ Monosaccharides were identified by GC/MS analysis of trimethylsilane derivatives.

**TABLE 3:** Linkage analysis of promonan EPS^*a*^

Residue	Relative peak area (%)
Terminal glucopyranosyl (t-Glc)	1.0
Terminal glucopyranosyl uronic acid (t-GlcA)	1.0
Terminal galactopyranosyl (t-Gal)	0.9
3-linked glucopyranosyl (3-Glc)	10.6
3-linked glucopyranosyl uronic acid (3-GlcA)	10.9
3-linked galactopyranosyl (3-Gal)	4.0
4-linked galactopyranosyl (4-Gal)	8.8
4-linked glucopyranosyl (4-Glc)	32.1
4-linked glucopyranosyl uronic acid (4-GlcA)	30.4
3,4-linked galactopyranosyl (3,4-Gal)	0.1
3,4-linked glucopyranosyl (3,4-Glc)	0.2
3,6-linked glucopyranosyl (3,6-Glc)	0.1

^*a*^ Semi-quantitative analysis of PMAAs prepared from promonan extracts.

### Targeted deletions of genes in the promonan cluster

**Fig 3:**
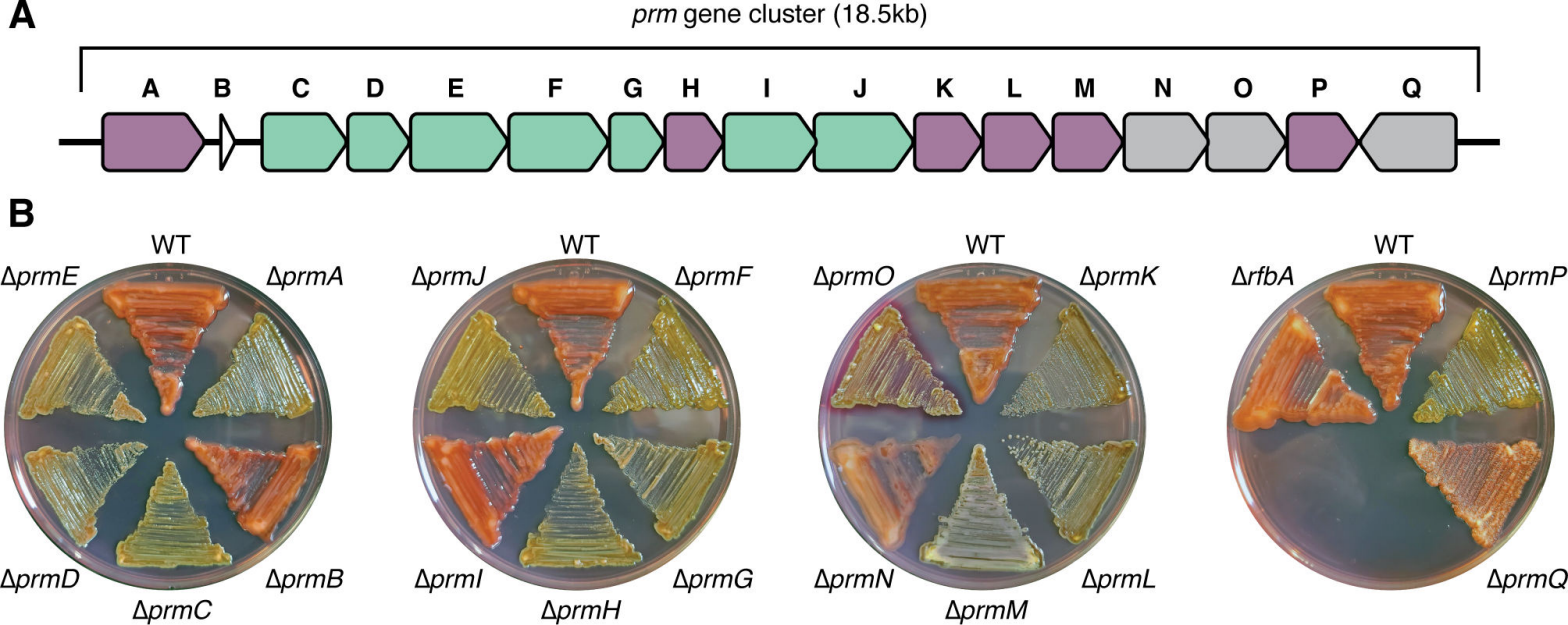
The promonan biosynthesis cluster. (**A**) Map of the *prm* cluster. Open-reading frames are colored by predicted function. Purple: monosaccharide incorporation; green: assembly/secretion; and gray: polysaccharide modification. (**B**) Morphologies of individual *prm* mutants on solid medium supplemented with Congo Red.

The promonan locus (Cluster A) contains 17 predicted open reading frames (BXU08_RS00350-BXU08_RS00430). Sixteen of the ORFs are arranged in the (+) direction on the chromosome, and a single ORF (BXU08_RS00430) sits at the 3′ end of the locus in the (−) direction (Fig. 3A). ORFs predicted to encode an initiating polyisoprenyl-phosphate hexose-phosphate transferase (PHPT), five GTs, and a full suite of polysaccharide assembly/secretion factors (Wza, CapB, Wzz, Wzx, and Wzy) make up a core set of polysaccharide biosynthesis genes. Additional genes for an outer membrane porin (OMP), an AAA+ family ATPase, an acyltransferase, and two glycosylhydrolases are also present along with a small (64 AA) ORF with no clear homology to any characterized protein families (Table 4). We predicted that this cluster contains the factors required for promonan biosynthesis and renamed these genes *prmA-Q* accordingly.

**TABLE 4:** Functional annotations and mutant phenotypes for promonan biosynthesis genes

Gene	Locus tag	Annotation	Phenotype
*prmA*	BXU08_RS00350	PHPT	EPS−
*prmB*	BXU08_RS00355	Hypothetical protein	Mucoid
*prmC*	BXU08_RS00360	Outer membrane beta-barrel protein	EPS−
*prmD*	BXU08_RS00365	SLBB domain polysaccharide export protein (Wza)	EPS−
*prmE*	BXU08_RS00370	AAA+ family ATPase	EPS−
*prmF*	BXU08_RS00375	Polysaccharide chain length regulator protein (Wzz/EpsF)	EPS−
*prmG*	BXU08_RS00380	CpsD/CapB family tyrosine protein kinase	EPS−
*prmH*	BXU08_RS00385	WecG/TagA/CpsF family glycosyltransferase	EPS−
*prmI*	BXU08_RS00390	Polysaccharide biosynthesis protein (Wzx)	Mucoid
*prmJ*	BXU08_RS00395	O-antigen ligase family protein (Wzy)	EPS−
*prmK*	BXU08_RS00400	Glycosyltransferase (GT2 family)	EPS−
*prmL*	BXU08_RS00405	Glycosyltransferase (GT2 family)	EPS−
*prmM*	BXU08_RS00410	Glycosyltransferase (GT2 family)	Mucoid, CR−
*prmN*	BXU08_RS00415	Glycosylhydrolase (GH10 family)	Mucoid
*prmO*	BXU08_RS00420	Acyltransferase (superfamily 3)	EPS−
*prmP*	BXU08_RS00425	Glycosyltransferase (GT2 family)	EPS−
*prmQ*	BXU08_RS00430	Glycosylhydrolase (GH16 family)	Rugose

We generated in-frame deletions for each of the 17 genes in the promonan cluster and assessed their EPS production phenotypes (Fig. 3B). Nearly all of the deletions led to a loss of Congo Red staining and promonan secretion, suggesting that these mutations caused an EPS− phenotype. The ∆*prmB* (hypothetical), ∆*prmI* (*wzx*), and ∆*prmN* (GH10) mutations had no effect on colony morphology. ∆*prmM* (GT) cells displayed a mucoid phenotype on agar plates but did not react with the Congo Red dye. The ∆*prmQ* (GH16) mutant had a non-mucoid phenotype that we classified rugose because it could be distinguished from other non-mucoid mutants by its dry, crusty appearance and its ability to react with Congo Red.

### Genetic complementation of mutants with altered promonan production

**Fig 4:**
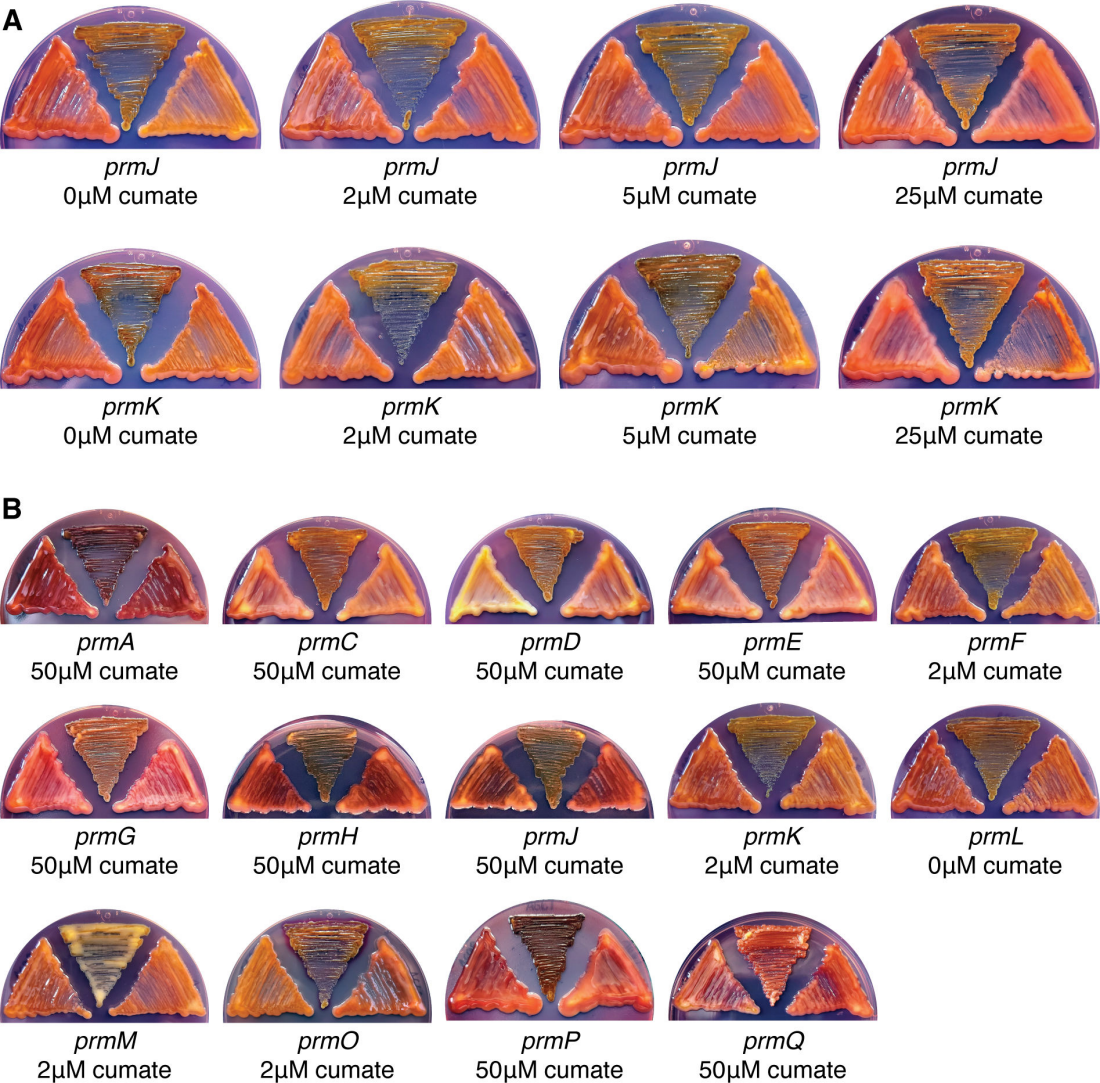
Genetic complementation of mutations affecting promonan production. All images show agar plates with wild-type LM7 on the left, the indicated deletion mutant containing a cumate-inducible form of the complementing gene on the right, and the respective deletion mutant containing an empty vector control in the center. (**A**) Low levels of *prmJ* induction give an intermediate complementation phenotype in the ∆*prmJ* mutant background, but higher levels of induction are needed for a full restoration of mucoidy. Complementation of the ∆*prmK* mutant is most effective at low inducer concentrations, while high levels of induction fail to restore mucoidy. (**B**) Complementation of promonan-associated phenotypes in individual *prm* mutants. The deletion mutant being complemented and the optimal inducer concentration are indicated below each image.

The phenotypic effects of deleting genes in the promonan cluster were confirmed through ectopic complementation. We used a cumate-inducible system developed by Kaczmarczyk et al. (27) to express *prm* genes from a plasmid in their respective deletion backgrounds. This system was reported to provide tight repression and highly tunable induction of heterologous gene expression in *Alphaproteobacteria*. We found that optimizing the expression levels of each gene was required for efficient complementation. Some genes, such as *prmJ* (described as *wzyA* above), required high levels of cumate induction to revert the phenotype of their associated deletion mutant. In other mutants, such as ∆*prmK*, inducing the complementing gene with low cumate concentrations restored the mucoid phenotype, but higher levels of induction abolished complementation (Fig. 4A). These results demonstrate that the expression levels of certain genes in the promonan pathway influence polysaccharide production. The disruption of polysaccharide production when some genes are over-expressed indicates that tuning expression levels of individual genes may be required to produce polysaccharides in recombinant systems. Figure 4B shows the complementation of all 14 mutants with altered mucoid phenotypes. In each case, transforming the deletion mutant with a plasmid containing a cumate-inducible form of the relevant gene and optimizing the induction conditions allowed for restoration of the wild-type colony morphology. These results confirm the EPS phenotypes assigned to individual mutants in the *prm* cluster (Table 4).

### Comparison of polysaccharide biosynthesis genes among *Alphaproteobacteria*

We compared the promonan EPS to polysaccharides produced by other *Alphaproteobacteria*. Certain isolates from the genus *Sphingomonas* produce acidic polysaccharides called sphingans that have gel-like properties reminiscent of our promonan extracts (28). The promonan gene cluster we identified does not show similarity to the conserved cluster for sphingan production (25). There is no recognizable synteny among core *wzy* genes (*wzy, wzx, wzy, wzz,* etc.). The promonan cluster also lacks ABC transporter genes and genes for dTDP-rhamnose synthesis. Finally, the presence of a predicted WecG-type GT gene (*prmH*) and two glycosylhydrolases (*prmN* and *prmQ*), all three of which are not present in sphingan gene clusters, further distinguishes the promonan cluster from the sphingan genes.

The presence of rhamnose in the repeating unit is a hallmark of sphingan EPSs. We did not detect rhamnose in the purified promonan extracts and could not identify a complete set of genes for dTDP-rhamnose biosynthesis in the LM7 genome. Homologs of *rfbA* and *rfbB* are present. *rfbD* appears to be present, but it codes for a protein that is fused to an additional beta-galactosidase domain. We could not identify *rfbC* in the LM7 genome. Though our genomic analysis suggests that LM7 is likely incapable of producing dTDP-rhamnose, we confirmed that rhamnose is not incorporated into promonan by deleting *rfbA* (BXU08_RS19140). The resulting ∆*rfbA* mutant showed wild-type colony morphology and Congo Red staining (Fig. 3B). Thus, the chemical compositions and genetic requirements for EPS production support the model that promonan and sphingan represent distinct EPS families.

**Fig 5:**
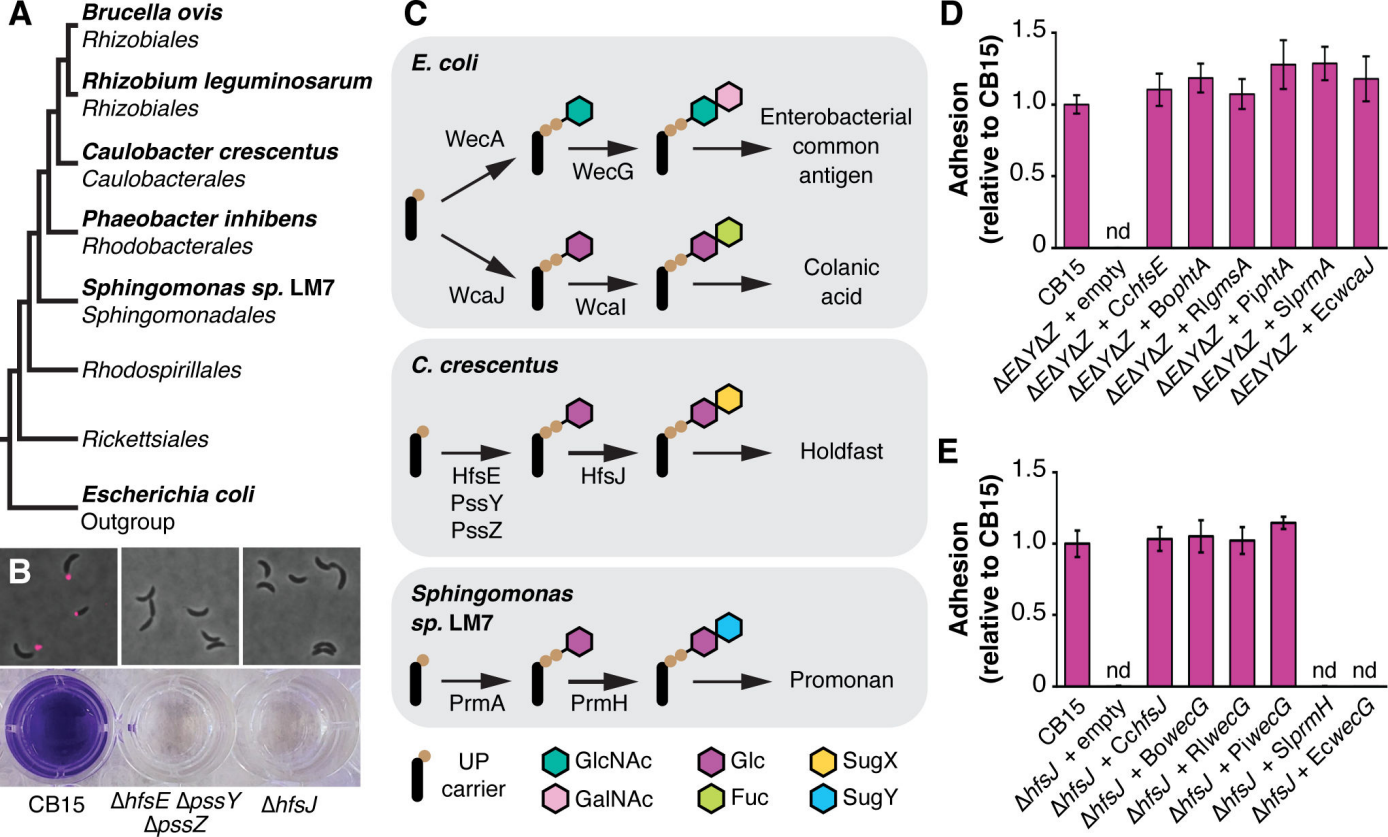
Conservation of polysaccharide biosynthesis genes in *Alphaproteobacteria.* (**A**) Schematic of *Alphaproteobacterial* phylogeny showing the relationship among isolates with putative polysaccharide adhesin pathways. (**B**) Phenotypes associated with holdfast production. Fluorescently labeled wheat germ agglutinin staining shows the loss of holdfast production in the *Caulobacter crescentus* ∆*hfsE* ∆*pssY* ∆*pssZ* and ∆*hfsJ* mutants. Crystal violet (CV) staining of cultures grown in microtiter plates shows the loss of surface adhesion in the ∆*hfsE* ∆*pssY* ∆*pssZ* and ∆*hfsJ* mutants. (**C**) Inferred activities for initiation and subsequent glycosyltransferase enzymes in four polysaccharide pathways. (**D**) CV staining assay testing the effect of introducing PHPT genes from various bacteria into the *C. crescentus* ∆*hfsE* ∆*pssY* ∆*pssZ* mutant. All tested genes appear capable of converting UndPP to UndPP-Glc. (**E**) CV staining assay testing the effect of introducing *wecG*-family GT genes from various bacteria into the *C. crescentus* ∆*hfsJ* mutant. *wecGs* from *Brucella ovis, Rhizobium leguminosarum,* and *Phaeobacter inhibens* can carry on the HfsJ reaction, while *wecG* from *Escherichia coli* (negative control) and *prmH* from *Sphingomonas* sp. LM7 cannot.

Many *Alphaproteobacteria* assemble adhesive polysaccharides at their cell poles (29–33), of which the holdfast polysaccharide from *Caulobacter crescentus* has been studied most extensively (34). We were intrigued by the identification of a WecG-type glycosyltransferase gene (*prmH*) in the promonan cluster because a WecG homolog (HfsJ) is also required for holdfast production (35). WecG-family GTs are thought to catalyze the committed step in select polysaccharide biosynthesis pathways by attaching a second sugar onto an UndPP-linked monosaccharide (36–38). We analyzed the genomes of a panel of *Alphaproteobacteria* that are known to produce holdfast-like adhesins. These bacteria, which span the phylogenetic diversity of *Alphaproteobacteria*, each contained a WecG-family GT gene (Fig. 5A). We predicted that the WecG-family GTs we identified might participate in a conserved pathway for producing holdfast-like adhesins.

We tested the conservation of holdfast-like EPS pathways by performing a series of cross-complementation experiments in *C. crescentus*. We reasoned that if an enzyme from one bacterium performs the same function as an enzyme in the holdfast pathway, introducing that gene into the corresponding *C. crescentus* deletion mutant should restore holdfast production. Polysaccharide biosynthesis begins when an initiator enzyme generates a lipid-linked monosaccharide. The production of undecaprenyldiphosphate-glucose (UndPP-Glc) by the redundant action of HfsE, PssY, and PssZ is thought to represent the initiation reaction in the holdfast pathway, and the product of this reaction likely serves as a substrate for HfsJ (39). Deleting the genes for these three initiators (∆*hfsE* ∆*pssY* ∆*pssZ*) causes a holdfast null phenotype (40) that can be scored using a crystal violet-based adhesion assay and visualized by staining holdfasts with fluorescently labeled wheat germ agglutinin (fWGA, Fig. 5B). The *wcaJ* gene from *Escherichia coli* codes for a undecaprenylphosphate glucose-phosphate transferase that produces UndPP-Glc for colonic acid biosynthesis (39), and we confirmed that introducing *wcaJ* into the ∆*hfsE* ∆*pssY* ∆*pssZ* mutant restored holdfast production. PHPT genes from each of the *Alphaproteobacteria* in our panel, including *prmA* from LM7, also restored holdfast production to the ∆*hfsE* ∆*pssY* ∆*pssZ* mutant, indicating that each of these bacteria encodes an enzyme that can support holdfast synthesis by producing UndPP-Glc (Fig. 5D).

Deleting the gene for the WecG-family GT *hfsJ* causes a holdfast null phenotype in *C. crescentus* (35). HfsJ is thought to generate a lipid-linked disaccharide by adding an unknown sugar to UndPP-Glc (produced by HfsE/PssY/PssZ). We introduced *wecG* homologs from our strain panel into the ∆*hfsJ* deletion to examine if they could restore holdfast production. *wecG*s from more derived (41) *Alphaproteobacterial* clades (*Rhizobiales, Caulobacterales,* and *Roseobacterales*) complemented the holdfast defect in ∆*hfsJ,* but *prmH* from LM7 did not. The *wecG* gene from *E. coli* codes for an enzyme that utilizes a different lipid-linked acceptor substrate (UndPP-GlcNAc) than *C. crescentus* HfsJ (UndPP-Glc) (36, 39), and we confirmed that introducing *EcwecG* did not restore holdfast production to the ∆*hfsJ* mutant (Fig. 5E). These results indicate that *wecG* homologs from representative *Rhizobiales, Caulobacterales,* and *Roseobacterales* can convert UndPP-Glc to a conserved UndPP-linked disaccharide, but that PrmH from LM7 likely converts UndPP-Glc to a different product. We conclude that a conserved pathway for holdfast-like adhesins exists in more derived clades of *Alphaproteobacteria,* but that promonan production likely represents a distinct biosynthetic pathway.

## DISCUSSION

The production of complex polysaccharides is nearly ubiquitous among bacteria. Despite the diversity of physical, chemical, and biological properties encompassed by bacterial polysaccharides, they remain underutilized in industrial settings. Our long-term goal is to harness the vast chemical space occupied by these glycans for the development of polymers with valuable properties. To this end, we began isolating bacteria from environmental sources and dissecting their polysaccharide biosynthesis pathways. An isolate from Lake Michigan called *Sphingomonas* sp. LM7 secretes an exopolysaccharide that we have named promonan. We identified a gene cluster that contains the factors required for promonan biosynthesis, defined the chemical composition of the polysaccharide, and showed that promonan represents an EPS that is distinct from previously characterized polysaccharide families.

Promonan biosynthesis clearly proceeds through a *wzx*/*wzy-*dependent mechanism. The *prm* locus contains genes for an initiating sugar phosphate transferase (*prmA*), five GTs (*prmH, prmK, prmL, prmM*, and *prmP*), and a full set of polymerization/export factors (*prmD, prmF, prmG, prmI,* and *prmJ*). *prmC* encodes an OMP that likely also plays a role in polysaccharide export (42, 43). Thus, 12 of the 17 genes in the *prm* cluster make up a core set of factors that should be sufficient to secrete a glycan with six or more residues in the repeating unit. Deleting any of the core genes causes a non-mucoid, EPS− phenotype, with the exception of *prmI* and *prmM. prmI* codes for a predicted Wzx-type repeating unit flippase that has no effect on colony morphology or EPS production. The apparent promiscuity of Wzx transporters involved in the assembly of the holdfast polysaccharide leads to genetic redundancy (40), and we predict that *prmI* is redundant with other glycolipid flippases in the LM7 genome. *prmM* codes for a predicted GT2-family glycosyltransferase. Deleting a GT that contributes to repeating unit assembly is expected to abolish polysaccharide production, but the ∆*prmM* mutant displays an unexpected phenotype. The colonies appear mucoid but do not take up Congo Red dye, suggesting that PrmM affects the properties of the EPS matrix but is not required for polysaccharide production.

Phenotypic analysis of *prm* genes that are not part of the predicted core pathway provides additional insight into the nuances of promonan biosynthesis. Deleting *prmB* (hypothetical protein) or *prmN* (GH10-family glycosylhydrolase) does not affect colony morphology or EPS production, indicating that these are dispensable for promonan production under our growth conditions. *prmE* encodes a predicted AAA+-type ATPase that is required for promonan production. ATPases from the bacterial tyrosine kinase (BYK) family participate in *wzy-*dependent polysaccharide production through a physical association with Wzz family co-polymerases (such as PrmF) (44–46). However, *prmG* codes for a bonified BYK protein, and the identification of PrmE as a second ATPase that is required for polysaccharide production suggests that the architecture of the polymerization/export machinery may be more complex in LM7 than in previously described systems. *prmO* codes for an OafA-family acyltransferase that is required for promonan production. The implications of an acyltransferase being required for promonan production are discussed below.

Deleting *prmQ* causes a unique phenotype characterized by colonies that produce a Congo Red reactive matrix but show a rugose morphology distinct from the smooth, mucoid appearance of wild-type colonies (Fig. 3B). *prmQ* codes for a predicted GH16-family glycosylhydrolase. These hydrolases are widespread across the tree of life. They display a range of carbohydrate-modifying activities, with the majority of characterized representatives having polysaccharide endo-glycosidase activity (47). PrmQ has a predicted signal sequence for export from the cytoplasm, and we predict that PrmQ directly hydrolyzes promonan during secretion, reducing the rigidity of the resulting polymers. The loss of such an activity would explain the rugose colony morphology seen in the ∆*prmQ* strain. A number of other polysaccharide biosynthesis clusters contain genes for glycosylhydrolases (48, 49), and we suspect that their presence reflects a need to accommodate cell growth and division by modifying the extracellular matrix.

While we have not yet determined a detailed chemical structure of promonan, the identification of Glc, Gal, and GlcA residues in their 3- and 4-linked forms indicates that the repeating unit contains six sugars in a linear, non-branching arrangement. A six-residue repeating unit might seem to align with the presence of six sugar transfer enzymes in the *prm* cluster. However, deleting the GT gene *prmM* does not alter colony mucoidy, but it causes cells to lose the ability to react with Congo Red dye. This phenotype suggests that *prmM* is not required for polysaccharide production but that its disruption alters the secreted matrix in some way. It is possible that PrmM incorporates a sugar into the growing repeating unit that is not required for downstream polymerization. Alternatively, deleting *prmM* could eliminate promonon production while activating the production of a separate, Congo Red insensitive extracellular matrix. Another intriguing aspect of promonan biosynthesis is the apparent requirement of an OafA-family acyltransferase gene (*prmO*) for polysaccharide production. The *oafA* gene from *Salmonella* Typhimurium confers acetylation of an abequose residue in the O-antigen of the O5 serotype, but disruption of *oafA* simply abolishes O-antigen acetylation rather than eliminating polysaccharide production (50). The non-mucoid, EPS− phenotype of the ∆*prmO* mutant suggests a different role for this gene in the promonan pathway. Acylation of a glycolipid intermediate by PrmO must be required for the assembly of a repeating unit that is competent for polymerization. Acetylation of an UndPP-linked disaccharide by WcaF is required for the assembly of the colonic acid repeating unit (51), and an OafA-family acyltransferase (UppZ) is required for unipolar polysaccharide assembly in *Agrobacterium tumefaciens* (49). We propose that the acylation of lipid-linked intermediates by OafA-family enzymes can be a critical step I in the assembly of repeating unit oligosaccharides. Our composition analysis did not detect acylated sugars in the promonan extracts, but future studies aimed at determining the chemical structures of intermediates in the pathway will help clarify the role of PrmO.

Analysis of functional redundancy among glycan assembly genes provided new insights into polysaccharide metabolism as a whole. Testing whether genes from different organisms could compensate for the loss of key enzymes in the *Caulobacter crescentus* holdfast pathway allowed us to dissect the substrate specificities of individual GTs. We identified a group of genes from diverse *Alphaproteobacteria* that appear to encode PHPT enzymes capable of generating UndPP-Glc. We could also distinguish the specificities of WecG-family GTs from these organisms by showing that only a subset could compensate for the loss of *hfsJ*. While the precise reaction catalyzed by HfsJ has yet to be determined, our method efficiently delineated GTs that share its specificity. This cross-complementation approach provided molecular evidence that a pathway for producing holdfast-like adhesins is conserved in more derived *Alphaproteobacterial* clades. More importantly, our results indicate that proteins with shared enzymatic activities but divergent sequences are interchangeable in polysaccharide biosynthesis pathways. Efforts to engineer the metabolism of other biopolymers have been hindered by the perturbation of protein-protein interactions and other unexpected disruptions when enzymatic functionalities are swapped genetically (52). *wzx*/*wzy*-dependent polysaccharide biosynthesis seems to present a truly modular system in which metabolic proteins can be interchanged without affecting pathway output.

Finally, this work highlights the extent to which bacterial polysaccharides remain under-sampled. Simply diluting water from a lake onto a standard growth medium allowed us to identify a family of EPSs that is distinct from previously characterized polysaccharides. Promonan is synthesized through a relatively simple *wzy-*dependent pathway that utilizes housekeeping sugars. Nonetheless, we identified five GTs with unknown linkage specificities, an acyltransferase that likely acts on a glycolipid, a matrix-modifying glycosylhydrolase, and an ATPase that has not previously been associated with polysaccharide assembly. Detailed biochemical analysis of these proteins will continue to illuminate the molecular basis for generating the vast complexity among bacterial glycans. These novel enzymatic functions can also be used to engineer unnatural chemical complexity into bacterial polysaccharides through combinatorial biosynthesis. Characterizing novel polysaccharide pathways in detail not only adds to a growing collection of diversity-generating enzymes, but streamlined assembly pathways like the one we identified in LM7 can also serve as scaffolds for the genetic engineering of novel polymers.

## MATERIALS AND METHODS

### Bacterial strains, growth conditions, and genetic manipulations

Strains and plasmids used in this study are listed in Tables 5 and 6. Standard polymerase chain reaction (PCR), restriction digestion, and Gibson assembly methods were used to develop plasmids. Strains, plasmids, primer sequences, and details of construction are available upon request. *E. coli* was cultured in LB medium at 37°C supplemented with 1.5% (wt/vol) agar, 60 mM diaminopimelic acid (DAP), 10 µg/mL tetracycline, and 50 µg/mL kanamycin when necessary. *C. crescentus* was grown at 30°C in the PYE medium supplemented with 1.5% (wt/vol) agar and 25 µg/mL kanamycin when necessary. Plasmids were introduced into *C. crescentus* by electroporation. *Sphingomonas* sp. LM7 was cultured at temperatures ranging from 18°C to 30°C in either PYE medium or M2 medium with glucose. Agar (1.5%, wt/vol), sucrose (3%, wt/vol), tetracycline (5 µg/mL), kanamycin (25 µg/mL), cumate (50 µM), Congo Red (20 µg/mL), and Coomassie Blue (10 µg/mL) were added to LM7 cultures when necessary. Plasmids were introduced into LM7 by conjugation with WM3064 as the donor strain.

**TABLE 5:** Plasmids used in this study

Plasmid	Description	Antibiotic	Reference
pKMW3	Plasmid for the delivery of barcoded himar transposon	Km	(53)
pNPTS138	Suicide plasmid for making unmarked deletions in *Sphingomonas* sp. LM7; carries *sacB* for counter-selection	Km	R. Alley (unpublished)
pDH298	To delete *prmJ;* Gibson cloning of fused upstream and downstream regions of BXU08_RS00395	Km	This work
pDH354	To delete *wzyB;* Gibson cloning of fused upstream and downstream regions of BXU08_RS19300	Km	This work
pDH1289	To delete *prmA;* Gibson cloning of fused upstream and downstream regions of BXU08_RS00350	Km	This work
pDH1290	To delete *prmB;* Gibson cloning of fused upstream and downstream regions of BXU08_RS00355	Km	This work
pDH1291	To delete *prmC;* Gibson cloning of fused upstream and downstream regions of BXU08_RS00360	Km	This work
pDH1292	To delete *prmD;* Gibson cloning of fused upstream and downstream regions of BXU08_RS00365	Km	This work
pDH1293	To delete *prmE;* Gibson cloning of fused upstream and downstream regions of BXU08_RS00370	Km	This work
pDH1294	To delete *prmF;* Gibson cloning of fused upstream and downstream regions of BXU08_RS00375	Km	This work
pDH1295	To delete *prmG;* Gibson cloning of fused upstream and downstream regions of BXU08_RS00380	Km	This work
pDH297	To delete *prmH;* Gibson cloning of fused upstream and downstream regions of BXU08_RS00385	Km	This work
pDH1296	To delete *prmI;* Gibson cloning of fused upstream and downstream regions of BXU08_RS00390	Km	This work
pDH1297	To delete *prmK;* Gibson cloning of fused upstream and downstream regions of BXU08_RS00400	Km	This work
pDH1298	To delete *prmL*; Gibson cloning of fused upstream and downstream regions of BXU08_RS00405	Km	This work
pDH1299	To delete *prmM;* Gibson cloning of fused upstream and downstream regions of BXU08_RS00410	Km	This work
pDH1300	To delete *prmN;* Gibson cloning of fused upstream and downstream regions of BXU08_RS00415	Km	This work
pDH1301	To delete *prmO;* Gibson cloning of fused upstream and downstream regions of BXU08_RS00420	Km	This work
pDH1302	To delete *prmP;* Gibson cloning of fused upstream and downstream regions of BXU08_RS00425	Km	This work
pDH331	To delete *prmQ;* Gibson cloning of fused upstream and downstream regions of BXU08_RS00430	Km	This work
pDH1303	To delete *rfbA;* Gibson cloning of fused upstream and downstream regions of BXU08_RS19140	Km	This work
pDH1248	pQF: pCM62 with cymR*, PQ5, and MCS for N- and C-terminal fusions to 3FLAG tag; Tcr	Tc	(27)
pDH1304	pQN: pQF derivative with N-terminal FLAG-tagging region removed; used for complementation experiments	Tc	This work
pDH1305	Gibson cloning of *prmH* into cumate-inducible plasmid pQN	Tc	This work
pDH1306	Gibson cloning of *prmQ* into cumate-inducible plasmid pQN	Tc	This work
pDH1307	Gibson cloning of *prmJ* into cumate-inducible plasmid pQN	Tc	This work
pDH1314	Gibson cloning of *prmC* into cumate-inducible plasmid pQN	Tc	This work
pDH1315	Gibson cloning of *prmD* into cumate-inducible plasmid pQN	Tc	This work
pDH1316	Gibson cloning of *prmE* into cumate-inducible plasmid pQN	Tc	This work
pDH1317	Gibson cloning of *prmF* into cumate-inducible plasmid pQN	Tc	This work
pDH1318	Gibson cloning of *prmG* into cumate-inducible plasmid pQN	Tc	This work
pDH1319	Gibson cloning of *prmK* into cumate-inducible plasmid pQN	Tc	This work
pDH1320	Gibson cloning of *prmL* into cumate-inducible plasmid pQN	Tc	This work
pDH1321	Gibson cloning of *prmM* into cumate-inducible plasmid pQN	Tc	This work
pDH1322	Gibson cloning of *prmO* into cumate-inducible plasmid pQN	Tc	This work
pDH1323	Gibson cloning of *prmP* into cumate-inducible plasmid pQN	Tc	This work
pDH1347	Gibson cloning of *prmA* into cumate-inducible plasmid pQN	Tc	This work
pDH118	pMT585; pMTLS4259: Integrating vector for xylose induceable expression of C-terminally GFP-tagged proteins in *Caulobacter*	Km	(54)
pDH444	Gibson cloning of *C. crescentus* CB15 CC_2425 into pDH118	Km	This work
pDH478	Gibson cloning of *R. leguminosarum* RL1661 into pDH118	Km	This work
pDH477	Gibson cloning of *Brucella ovis* BOV_0775 into pDH118	Km	This work
pDH445	Gibson cloning of *Phaeobacter inhibens* PGA1_262p00380 into pDH118	Km	This work
pDH1288	Gibson cloning of *Sphingomonas* sp*.* LM7 BXU08_RS00350 into pDH118	Km	This work
pDH476	Gibson cloning of *E. coli* wcaJ into pDH118	Km	This work
pDH124	Gibson cloning of *C. crescentu*s CB15 CC_0095 into pDH118	Km	This work
pDH125	Gibson cloning of *B. ovis* BOV_A0095 into pDH118	Km	This work
pDH128	Gibson cloning of *R. leguminosarum* RL0741 into pDH118	Km	This work
pDH295	Gibson cloning of *Sphingomonas* sp. LM7 BXU08_RS00385 into pDH118	Km	This work
pDH155	Gibson cloning of *E. coli wecG* into pDH118	Km	This work

**TABLE 6:** Strains used in this study

Strain	Organism	Genotype	Description	Source
DH173	*Sphingomonas* sp. LM7	LM7	Wild type	This work
DH313	*Sphingomonas* sp. LM7	Δ*prmJ*	In-frame deletion of BXU08_RS00395	This work
DH366	*Sphingomonas* sp. LM7	∆*wzyB*	In-frame deletion of BXU08_RS19300	This work
DH1272	*Sphingomonas* sp. LM7	Δ*prmA*	In-frame deletion of BXU08_RS00350	This work
DH1273	*Sphingomonas* sp. LM7	Δ*prmB*	In-frame deletion of BXU08_RS00355	This work
DH1274	*Sphingomonas* sp. LM7	Δ*prmC*	In-frame deletion of BXU08_RS00360	This work
DH1275	*Sphingomonas* sp. LM7	Δ*prmD*	In-frame deletion of BXU08_RS00365	This work
DH1276	*Sphingomonas* sp. LM7	Δ*prmE*	In-frame deletion of BXU08_RS00370	This work
DH1277	*Sphingomonas* sp. LM7	Δ*prmF*	In-frame deletion of BXU08_RS00375	This work
DH1278	*Sphingomonas* sp. LM7	Δ*prmG*	In-frame deletion of BXU08_RS00380	This work
DH314	*Sphingomonas* sp. LM7	Δ*prmH*	In-frame deletion of BXU08_RS00385	This work
DH1279	*Sphingomonas* sp. LM7	Δ*prmI*	In-frame deletion of BXU08_RS00390	This work
DH1280	*Sphingomonas* sp. LM7	Δ*prmK*	In-frame deletion of BXU08_RS00400	This work
DH1281	*Sphingomonas* sp. LM7	Δ*prmL*	In-frame deletion of BXU08_RS00405	This work
DH1282	*Sphingomonas* sp. LM7	Δ*prmM*	In-frame deletion of BXU08_RS00410	This work
DH1283	*Sphingomonas* sp. LM7	Δ*prmN*	In-frame deletion of BXU08_RS00415	This work
DH1284	*Sphingomonas* sp. LM7	Δ*prmO*	In-frame deletion of BXU08_RS00420	This work
DH1285	*Sphingomonas* sp. LM7	Δ*prmP*	In-frame deletion of BXU08_RS00425	This work
DH345	*Sphingomonas* sp. LM7	Δ*prmQ*	In-frame deletion of BXU08_RS00430	This work
DH1286	*Sphingomonas* sp. LM7	Δ*rfbA*	In-frame deletion of BXU08_RS19140	This work
DH103	*C. crescentus* CB15	CB15	Wild type	(55)
DH433	*C. crescentus* CB15	∆*pssY* ∆*pssZ* ∆*hfsE*	In-frame deletion of CC_0166, CC_2384, and CC_2425	(40)
DH450	*C. crescentus* CB15	∆pssY∆pssZ∆hfsE/pDH118	pDH118 integrated at the xylose locus of DH433	This work
DH452	*C. crescentus* CB15	∆pssY∆pssZ∆hfsE/pXyl::Cc*hfsE*	pDH444 integrated at the xylose locus of DH433	This work
DH489	*C. crescentus* CB15	∆pssY∆pssZ∆hfsE/pXyl::Bo*phtA*	pDH478 integrated at the xylose locus of DH433	This work
DH488	*C. crescentus* CB15	∆pssY∆pssZ∆hfsE/pXyl::RlvgmsA	pDH477 integrated at the xylose locus of DH433	This work
DH453	*C. crescentus* CB15	∆pssY∆pssZ∆hfsE/pXyl::PiphtA	pDH445 integrated at the xylose locus of DH433	This work
DH1287	*C. crescentus* CB15	∆pssY∆pssZ∆hfsE/pXyl::Sl*prmA*	pAG42 integrated at the xylose locus of DH443	This work
DH487	*C. crescentus* CB15	∆pssY∆pssZ∆hfsE/pXyl::Ec*wcaJ*	pDH476 integrated at the xylose locus of DH433	This work
DH105	*C. crescentus* CB15	∆hfsJ; FC1974	In-frame deletion of CC_0095	(35)
DH141	*C. crescentus* CB15	∆*hfsJ/pDH118*	pDH118 integrated at the xylose locus of DH105	This work
DH132	*C. crescentus* CB15	∆hfsJ/pXyl::Cc*hfsJ*	pDH124 integrated at the xylose locus of DH105	This work
DH133	*C. crescentus* CB15	∆hfsJ/pXyl::Bo*wecG*	pDH125 integrated at the xylose locus of DH105	This work
DH136	*C. crescentus* CB15	∆hfsJ/pXyl::Rl*wecG*	pDH128 integrated at the xylose locus of DH105	This work
DH355	*C. crescentus* CB15	∆hfsJ/pXyl::Sl*prmH*	pDH295 integrated at the xylose locus of DH105	This work
DH160	*C. crescentus* CB15	∆hfsJ/pXyl::E*cwecG*	pDH155 integrated at the xylose locus of DH105	This work

Gene deletions were generated using a two-step approach with *sacB-*based counterselection. To generate plasmids for making deletions, ~500 bp fragments from upstream and downstream of the target genes were fused and inserted into the SpeI/HindIII site of pDH100. For all but two of the deletions, the first and last 12 nt of the target ORF were included in the deletion vector to minimize polar effects. The *prmP* and *prmQ* ORFs overlap by 29 bp. For both the *prmP* and *prmQ* deletions, 12 bp on the 5′ end of the target ORF and 42 bp on the 3′ end of the target ORF were retained in the deletion construct to avoid deleting part of the adjacent gene. Deletion plasmids were introduced into LM7 by electroporation. Primary integrants were selected on PYE supplemented with kanamycin, patched onto a second PYE plate containing kanamycin, and re-grown overnight. Cells from purified primary integrant colonies were inoculated into PYE medium, grown overnight, and serially diluted on PYE plates containing sucrose. Sucrose-resistant colonies were screened for sensitivity to kanamycin. KanS colonies were then screened by PCR to identify strains containing the appropriate deletion.

### Isolation of *Sphingomonas* sp. LM7

Water from Lake Michigan was collected near the shore of Promontory Point in Chicago, IL, USA, using a plastic beer pitcher attached to the end of a 10 ft metal pole. The water was serially diluted, plated on PYE agar, and incubated at 30°C. A single, yellow colony was isolated for further study.

### Genome sequencing

Genomic DNA isolated from LM7 was sequenced on a PacBio RS II instrument at the University of Wisconsin–Milwaukee Great Lakes Genomics Center. Genome assembly using HGAP3 (56) yielded a single, circular contig based on ~228× coverage. The genome was deposited under NCBI RefSeq NZ_CP019511.1.

### Transposon mutagenesis and analysis of mutants with altered colony morphology

*Sphingomonas* sp. LM7 was mutagenized with a modified mariner transposon. WM3064 cells carrying pKMW3 (53) were mixed with LM7 cells at a ratio of approximately 1:4. The mixture was spotted on PYE plates containing DAP and incubated overnight at room temperature. Cells from the conjugation reaction were resuspended in PYE, diluted appropriately, and spread onto 150 mm plates containing M2 medium with 2% (wt/vol) glucose and kanamycin. Plates were incubated for 5 days at room temperature. Mutants were screened visually for changes in colony morphology. Mutants of interest were isolated and patched onto PYE agar containing kanamycin for further study.

Mutations were mapped using a two-step, arbitrary PCR strategy. For these reactions, 2× GoGreen Master Mix (Promega) was used. A primer containing a sequence from the 3′ end of the transposon cassette (U1 fw: 5′-GATGTCCACGAGGTCTCT-3′) and a primer containing the M13 priming sequence fused to a random heptamer (M13-N_7_: 5′ TGTAAAACGACGGCCAGTNNNNNNN-3′) were used to amplify the junction between the transposon and genomic insertion site. The conditions for this reaction were as follows: 95°C for 2 min, 35 cycles of 95°C for 30 s, 38°C for 30 s, and 72°C for 1 min, followed by a 5-min hold at 72°C. The reaction was treated with ExoSap-IT (ThermoFisher), and a second, nested amplification reaction was then performed using this ExoSap-treated product as a template. A different region from the 3′ end of the transposon cassette (U2out: 5′ CGTACGCTGCAGGTCGAC-3′) and the M13 priming sequence served as primers for this second reaction. Reactions were incubated at 95°C for 2 min, and a 40-cycle touchdown program of 95°C for 30 s, a 30 s annealing step, and 72°C for 1 min was applied followed by a 5-min incubation at 72°C. The annealing step was performed at 68°C for the first cycle and decreased by 0.5°C with each cycle. The nested reaction products were treated with ExoSap and subjected to Sanger sequencing to identify the genomic sites of transposon insertion.

### Isolation of promonan EPS

Overnight cultures of *Sphingomonas* sp. LM7 grown in PYE were used to inoculate 1 L of M2 medium supplemented with 2% (wt/vol) D-glucose. Cultures were grown at 18°C for 72 h with shaking at 200 rpm. Cells were removed from the culture broth by centrifugation at 8,000 × *g*, and the resulting supernatant was transferred to fresh tubes. Two volumes of ethanol were added to the spent medium, and the mixture was incubated overnight at 4°C. Precipitate was harvested by centrifuging for 1 h at 8,000 × *g*. After discarding the supernatant, the gel-like insoluble fraction was resuspended in water. Tris-HCl, pH 7.4, MgCl_2_, and turbonuclease were added to final concentrations of 10 mM, 2 mM, and 1 µg/mL, respectively. The nuclease digest was incubated overnight at 37°C. Tris-HCl, pH 8.5, and proteinase K were then added to final concentrations of 25 mM and 100 µg/mL, respectively, and the digest was again allowed to proceed overnight at 37°C.

An equal volume of aqueous phenol was added to the extraction. The mixture was homogenized thoroughly, 10–15 drops of chloroform were added, and the layers were allowed to separate for 30 min. The upper aqueous layer was then transferred to dialysis tubing (8 kDa cutoff) and was dialyzed against deionized water for 72 h. The dialysate was transferred to fresh deionized water every 24 h. The resulting extracts were transferred to glass tubes, incubated at −20°C overnight, and lyophilized to dryness.

### Monosaccharide composition analysis

The analysis was performed by combined gas chromatography-mass spectrometry (GC-MS) of the TMS methyl glycoside derivatives produced from the sample by acidic methanolysis. First, a 1 mg/mL solution of each sample was made. Of this solution, 200 µL was combined with 2 µL of a 10 mg/mL stock solution of myo-inositol, and the resulting mixture was lyophilized. To the dried samples, 15 drops of 1 M methanolic-HCl were added. The samples were incubated for 18 h at 80°C. The following day, the samples were removed from the heating block and cooled to room temperature. Once cooled, they were dried with dry nitrogen gas, during which five drops of MeOH were added every 3 min. Next, the samples were N-acetylated with eight drops of MeOH, four drops of pyridine, and four drops of acetic anhydride, and the mixture was incubated at 100°C for 30 min. Once cooled to room temperature, the samples were dried with dry nitrogen gas. Five drops of MeOH were added after 5 min, and after complete drying, 10 drops of Tri-Sil HTP reagent were added to each sample, followed by incubation at 80°C for 20 min. After evaporation of the solvent, 100 µL of hexane was added to each sample. The samples were briefly vortexed and centrifuged and then transferred to GC vials. For analysis, 1 µL was injected. GC-MS analysis of the TMS methyl glycosides was performed on an AT 7890A GC interfaced to a 5975B MSD, using an EC-1 fused silica capillary column (30 m × 0.25 mm ID). The temperature gradient involved an initial 2-min hold at 80°C, a 20°C/min ramp to 140°C, a 2-min hold at 140°C, a 2°C/min ramp to 200°C, a 30°C/min ramp to 250°C, and a final 5-min hold at 250°C.

### Glycosyl linkage analysis

The identification of glucuronic acid in the promonan extracts complicated the analysis of PMAAs because uronic acids diminish solubility in dimethylsulfoxide, leading to poor results. We deployed a recently developed method (26) whereby the acidic polysaccharide is first acetylated in an ionic liquid to increase solubility, leading to vastly improved recoveries.

Samples were acetylated by dissolving in 300 µL of the ionic liquid 1-ethyl-3-methylimidazodium acetate and sonicating them to fully dissolve. Once fully dissolved, 500 µL of acetic anhydride and 50 µL of 1-methylimidazole were added. The samples were then stirred for 10 min. After stirring, 2 mL of dichloromethane (DCM) and 2 mL of H_2_O were added. Each sample was vortexed and centrifuged briefly, and then the aqueous layer was carefully discarded. Each sample contained a white interphase. All of the water was not removed so as not to disturb this phase. The extraction was repeated four times. On the final wash, the organic (DCM) layer was removed and dried down with dry nitrogen. Once the DCM was completely removed, the samples were lyophilized. After this, the samples were dissolved in 300 µL of dimethylsulfoxide (DMSO). Next, 300 µL of potassium methylsulfinylmethylide in DMSO (~1 M) was added to each sample, which was stirred for 1 h. The samples were then placed in an ice bath until the solution solidified. A volume of 100 µL of iodomethane was then added at a rate that prevented the solution from boiling. Once the solution color changed from dark brown to light brown, another 100 µL of iodomethane was added, and the samples were stirred for about 25 min to ensure full permethylation of the uronic acids. Another DCM extraction was performed, and the DCM layer was dried with dry nitrogen after the final wash. The samples were then lyophilized overnight before continuing with reduction.

To begin reduction, 300 µL of a 10 mg/mL solution of lithium aluminum deuteride (LiAlD_4_) in tetrahydrofuran was added to the dried sample, which was then incubated at 80°C for 4 h. The reaction was then neutralized with two drops of acetic acid (AcOH). Five drops of MeOH were added to each. The samples were then dried with dry nitrogen gas. Once fully dry, 10 drops of 9:1 MeOH:AcOH were added, and the samples were dried again. Again, 10 drops of 9:1 MeOH:AcOH were added and dried one more time. Next, 10 drops of MeOH were added and dried with dry nitrogen, and this step was repeated two times. The samples were redissolved in 2 mL of H_2_O, transferred to 6 kDa dialysis bags, and dialyzed against two exchanges of DI water per day for 2 days. Following dialysis, the samples were permethylated according to the following procedure.

Sodium hydroxide (NaOH) base was prepared according to the procedure of Anumula and Taylor (57), and of this, 300 µL was added to all samples. The sample containing base was stirred for 15 min. A volume of 150 µL of iodomethane was added, and the mixture was stirred for 25 min. The steps adding base and iodomethane were repeated one more time to ensure that the samples were fully methylated. Once mixing was completed, 2 mL of dichloromethane and 2 mL H_2_O were added. The tube was vortexed and centrifuged briefly and then the upper (aqueous) layer was removed. This step was repeated four more times. After the final wash, the DCM layer was removed into a separate tube and dried with dry nitrogen gas.

The samples were hydrolyzed in 2 M trifluoroacetic acid (TFA) for 2 h in a sealed tube at 120°C. The hydrolyzed samples were cooled to room temperature, and the TFA was evaporated using dry nitrogen gas. Once fully dry, 10 drops of isopropanol (IPA) were added. The samples were dried again, and the addition and drying of IPA were repeated two times. On the last addition of IPA, a 10 mg/mL solution of NaBD_4_ and 1 M NH_4_OH was made with 9.48 mg of NaBD_4_ and 948 µL of NH_4_OH. Of the NaBD_4_ solution, 400 µL was added to each sample. The samples were left to incubate for reduction overnight. The following day, the basic reaction was neutralized with three drops of acetic acid. Five drops of MeOH were added to each. The samples were then dried with dry nitrogen gas. Once fully dry, 10 drops of 9:1 MeOH:AcOH was added, and the samples were dried again. Then, 10 drops of 9:1 MeOH:AcOH was added and dried one more time. Next, 10 drops of MeOH were added and dried with dry nitrogen, and this step was repeated two times. The samples were then O-acetylated with 250 µL of acetic anhydride and 230 µL of TFA and incubated at 50°C for 10 min. Once cooled to room temperature, a DCM extraction was done by adding 2 mL of DCM and 2 mL of H_2_O to each sample. The mixture was briefly vortexed and centrifuged. The aqueous layer was carefully decanted, and the process was repeated four times. On the final wash, the DCM layer was removed into a separate tube and dried with dry nitrogen. Five drops of DCM were then added and transferred to GC vials. A volume of 1 µL of each was injected into the GC-MS for analysis. The resulting linkages were analyzed on an Agilent 7890A GC interfaced to a 5975C MSD, electron impact ionization mode. Separation of natural monosaccharides was performed on a 30-m Supelco SP-2331 bonded phase fused silica capillary column. The column was subjected to a 1 min initial hold at 60°C. The temperature was then increased to 170°C over 4 min, raised to 235°C over 16.5 min, held at 235°C for 2 min, and finally raised to 240°C over 1.4 min followed by a 12 min final hold at 240°C.

### Crystal violet staining assay

Crystal violet staining analysis of adhesion in *C. crescentus* strains was performed as described previously (58). Overnight cultures grown in PYE were normalized to an OD_600_ of 0.5, and 1 µL of normalized culture was added to 450 µL of PYE in a 48-well microtiter plate. Plates were incubated for 18–20 h at 30°C with shaking at 155 rpm. The culture broth was discarded, the wells were washed thoroughly with tap water, and 500 µL of 0.01% (wt/vol) crystal violet was added to each well. The plate was shaken at 155 rpm with the dye for 5 min before the dye was discarded, and the wells were again washed thoroughly with tap water. Retained dye was then dissolved in 500 µL of ethanol by shaking for 5 min at 155 rpm. Absorbance at 575 nm was measured in each well.

### Visualization of holdfast production

Overnight cultures of *C. crescentus* strains of interest were grown overnight, diluted into fresh PYE, and allowed to reach the mid-log phase (OD_600_ = 0.4–0.5). A volume of 200 µL of culture was added to a clean tube, 1 µL of Alexa647-conjugated fWGA was added from a 2 mg/mL stock, and the labeling reaction was incubated at room temperature for 5 min in the dark. A volume of 1 mL of sterile water was added to the tube, and the mixture was centrifuged for 2 min at 6,000 × *g* to sediment cells. The supernatant was discarded, cells were resuspended in the residual liquid, and 1 µL was spotted onto 1% agarose pads. Microscopy was performed using a Nikon Ti-E inverted microscope equipped with an Orca Fusion BT digital CMOS camera (Hamamatsu). Fluorescence images were collected using a Prior Lumen 200 metal halide light source and a mCherry-specific filter set (Chroma).

## Data Availability

Reads and metadata from the LM7 genome sequencing project can be accessed through NCBI BioProject PRJNA363095. Sanger sequencing reads from transposon insertion mapping, microscopy images, and any other data collected during this work have been archived and are available upon request.
